# Comparison of time-dependent effects of (+)-methamphetamine or forced swim on monoamines, corticosterone, glucose, creatine, and creatinine in rats

**DOI:** 10.1186/1471-2202-9-49

**Published:** 2008-05-30

**Authors:** Nicole R Herring, Tori L Schaefer, Peter H Tang, Matthew R Skelton, James P Lucot, Gary A Gudelsky, Charles V Vorhees, Michael T Williams

**Affiliations:** 1 Division of Neurology, Cincinnati Children's Research Foundation and University of Cincinnati College of Medicine, Cincinnati, Ohio, USA; 2 College of Pharmacy, University of Cincinnati, Cincinnati, Ohio, USA; 3 Department of Pharmacology and Toxicology, Wright State University, Dayton, Ohio, USA

## Abstract

**Background:**

Methamphetamine (MA) use is a worldwide problem. Abusers can have cognitive deficits, monoamine reductions, and altered magnetic resonance spectroscopy findings. Animal models have been used to investigate some of these effects, however many of these experiments have not examined the impact of MA on the stress response. For example, numerous studies have demonstrated (+)-MA-induced neurotoxicity and monoamine reductions, however the effects of MA on other markers that may play a role in neurotoxicity or cell energetics such as glucose, corticosterone, and/or creatine have received less attention. In this experiment, the effects of a neurotoxic regimen of (+)-MA (4 doses at 2 h intervals) on brain monoamines, neostriatal GFAP, plasma corticosterone, creatinine, and glucose, and brain and muscle creatine were evaluated 1, 7, 24, and 72 h after the first dose. In order to compare MA's effects with stress, animals were subjected to a forced swim test in a temporal pattern similar to MA administration [i.e., (30 min/session) 4 times at 2 h intervals].

**Results:**

MA increased corticosterone from 1–72 h with a peak 1 h after the first treatment, whereas glucose was only increased 1 h post-treatment. Neostriatal and hippocampal monoamines were decreased at 7, 24, and 72 h, with a concurrent increase in GFAP at 72 h. There was no effect of MA on regional brain creatine, however plasma creatinine was increased during the first 24 h and decreased by 72 h. As with MA treatment, forced swim increased corticosterone more than MA initially. Unlike MA, forced swim reduced creatine in the cerebellum with no change in other brain regions while plasma creatinine was decreased at 1 and 7 h. Glucose in plasma was decreased at 7 h.

**Conclusion:**

Both MA and forced swim increase demand on energy substrates but in different ways, and MA has persistent effects on corticosterone that are not attributable to stress alone.

## Background

The psychostimulant methamphetamine (MA) is an addictive drug that has surged in popularity in the past 15 years. In the United States, 4.4% of 12^th ^graders and ~7.2% of 29–30 year olds reported life-time MA use in 2006 [1,2]. MA intoxication heightens attention and decreases fatigue, appetite, and anxiety along with stimulating the sympathetic nervous system, resulting in tachycardia, hypertension, diaphoresis, tachypnea, and hyperthermia [3], symptoms also associated with stressful situations. Consistent with a stress-like response, MA exposure also produces elevated cortisol in humans [4]. In addition, acute MA intoxication often results in increased serum creatinine, the metabolite of creatine, which may be due to rhabdomyolysis with acute renal failure [5]. Chronic use of MA produces detrimental alterations in the brain. For example, reductions of brain monoamines and associated reuptake transporters are observed in abusers [3,6,7]. Likewise, magnetic resonance spectroscopy studies have shown reduced total creatine (α-methyl-guanidinoacetic acid) in the basal ganglia after MA exposure in adults but increased creatine in school-aged children who were exposed to MA *in utero *[7]. Concurrent with the various neurochemical changes that occur following chronic abuse of MA, various studies have shown that humans have long-term learning and memory deficits, even after a period of abstinence. The mechanism(s) underlying the learning and memory deficits following MA are unknown, although stress-related pathways may be involved since increased cortisol [8] has been shown to affect cognitive function. Furthermore, the creatine system may be involved in these learning deficits, since it has been shown that learning and memory are affected by reductions in creatine in the brain [9].

MA treatment in rats (single-day, 5–10 mg/kg repeated at 2–3 h intervals) produces monoamine and associated transporter reductions similar to those seen in humans [10,11]. In addition, MA induces astrogliosis (increased glial fibrillary acidic protein (GFAP)), argyrophilia, and Fluoro-Jade staining [12,13], all indicators of neurotoxicity. Similar to humans, corticosterone and adrenocorticotrophin hormone (ACTH) are elevated after MA exposure in rats [14,15], although a time course of corticosterone increase in rats following MA is unknown. MA is also pyrogenic and at sufficient doses induces hyperthermia. MA-induced hyperthermia may play a role in the neurotoxic effects of the drug [12].

Because of the increased activation of stress-related pathways following MA administration and the lack of a more immediate profile (e.g., between 1–24 h following the first dose) for these changes, the first objective of this study was to examine various physiological parameters during and shortly following MA treatment. Corticosterone was selected as a marker of hypothalamic-pituitary-adrenal (HPA) axis activation, glucose as a marker of homeostasis, and levels of monoamines in the brain were assessed because of the well-known effect of MA on these neurotransmitters. Creatine in the brain in various regions and creatinine in plasma were assessed because the human literature demonstrates lasting changes in the creatine system following MA abuse and acute changes in creatinine during MA intoxication. The creatine system is a significant source of metabolic energy by providing a substrate for ATP-regeneration and storage in the brain [16]. A later time-point following MA (i.e., 72 h) was added to corroborate previously reported effects on markers of neurotoxicity, i.e., increased GFAP and decreased levels of dopamine in the neostriatum [13]. Once a profile of effects during the first 24 h was established for MA, we compared that profile to one obtained using forced swim to determine if MA produced unique effects that were distinguishable from a stressor. Accordingly, we conducted a second experiment in which we investigated the effects of a stressor (forced swim) in an experimental design similar to that used to investigate the effects of MA.

In order to compare the effects of MA and stress, we wanted a stressor known to produce a large increase in corticosterone comparable to that caused by MA. Kirby et al. [17] showed that forced swim (FS) for 30 min increased corticosterone immediately following exposure more than tail pinch, cold, immobilization, or forced motor activity (rotarod). In addition, FS was selected because MA-treated animals display hyperactivity during drug treatment and animals require active movement during swimming (whereas animals immobilized or restrained do not). Forced swim for 30 min has also been shown to affect the 5-HT system by increasing 5-HT (up to 3 h after the swim) and decreasing 5-HIAA in the striatum and in the hippocampus (up to 90 min after the swim) [17].

## Results

### Experiment-1

In Experiment 1, animals were given 10 mg/kg MA or an equivalent volume of saline (SAL), four times on a single day at 2 h intervals. Two cohorts of animals were used.

#### Body Temperatures

**Figure 1:**
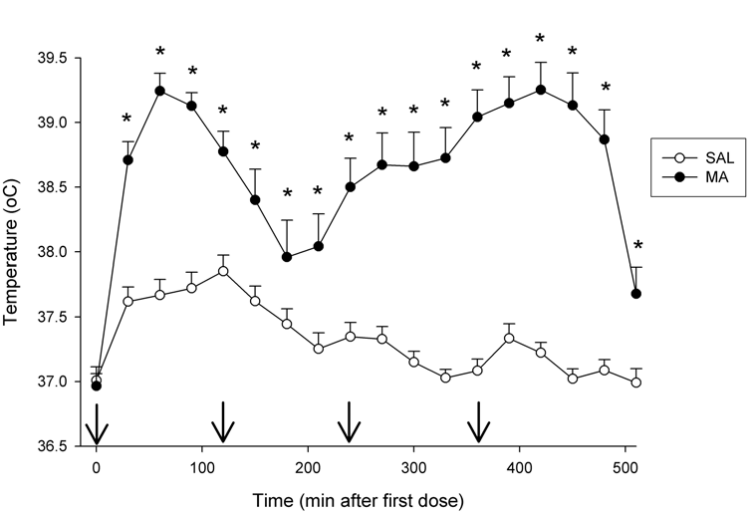
**Body temperatures of animals in Experiment 1 (mean ± SEM). **No differences in initial temperatures were observed (Time = 0); however, MA produced significant increases in body temperature during subsequent temperature collection times. Arrows denote injection times. *p ≤ 0.05 *vs *SAL.

Body temperatures were collected immediately prior to MA and every 30 min during and after MA treatment until 2 h following the first dose. There were no differences in body temperatures among groups prior to drug treatment. There were significant main effects of Treatment, F(1, 47) = 63.17, p < 0.0001, and Time (p < 0.0001), as well as the interaction of Treatment × Time, F(17, 799) = 8.12, p < 0.0001, on post-drug body temperatures. Examination of the Treatment × Time interaction revealed that animals treated with MA began showing hyperthermia relative to SAL-treated animals beginning ~30 min after the first dose (p < 0.0001) and this continued for the remainder of the temperature collection period (30–570 min, p ≤ 0.01; Fig. 1). The decline of body temperature in MA-treated animals that reached a nadir at 180 min was caused by slight overcooling of the first cohort of MA-treated animals. The cooling procedure was used to ensure that animals did not die because of hyperthermia, but it was insufficient to protect animals from MA-induced decreases in monoamines or neurotoxicity. Based on the rapid decrease in temperature with some of the animals in the first cohort, body temperatures were monitored more closely and cooling was discontinued when body temperatures began to decline in the second cohort. Examination of the outcome measures, however, revealed no differences between cooled and non-cooled MA-treated animals, indicating that this transient temperature reduction did not alter the effects of MA and succeeded in protecting animals from hyperthermia-induced death.

#### Body Weights

In addition to body temperatures, body weights were collected at predetermined time intervals. There were no initial differences in body weights between treatment groups (not shown). At 24 h following the first dose, MA-treated animals weighed significantly less than SAL-treated animals, F(1, 35) = 7.61, p < 0.01 (not shown). There were no differences between MA- and SAL-treated animals at 48 or 64 h (not shown).

#### Corticosterone

**Figure 2:**
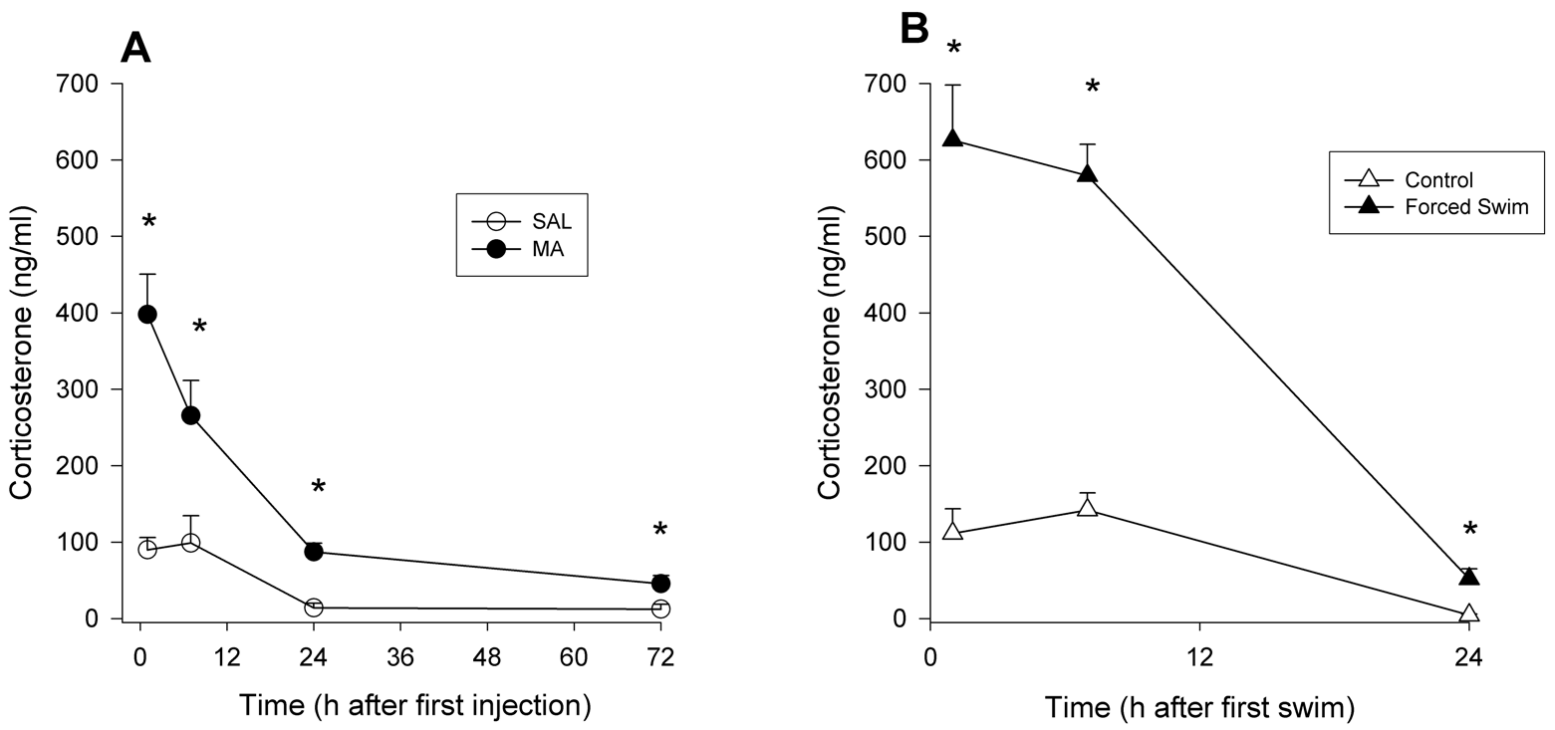
**Corticosterone levels in plasma following methamphetamine treatment in Experiment 1 (A) or forced swim in Experiment 2 (B) (mean ± SEM).** MA-treated animals demonstrated increased corticosterone up to 72 h after the first dose compared to SAL-treated animals (A). Likewise, forced swim animals demonstrated increased corticosterone levels up to 24 h (the last time point examined) after the beginning of the first swim (B). *p ≤ 0.05 *vs *SAL.

Plasma was collected from groups of animals at 1, 7, 24, and 72 h after the first dose of MA and assessed for corticosterone, glucose, and creatinine. Corticosterone levels were significantly affected by MA treatment. There were effects of Treatment, F(1, 76) = 73.33, p < 0.0001, Time (p < 0.0001), and the interaction of Treatment × Time, F (3, 76) = 11.92, p < 0.0001. Examination of the interaction showed that 1 h after the first dose, corticosterone levels were increased in MA-treated animals compared to SAL-treated animals. Corticosterone levels in MA-treated animals declined thereafter, but remained significantly elevated compared to SAL-treated animals at 7, 24, and 72 h (Fig. 2A).

#### Glucose

**Figure 3:**
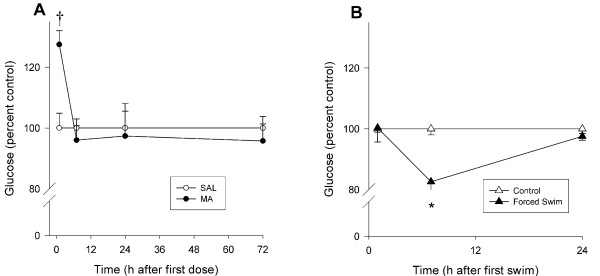
**Glucose levels in plasma expressed as a percent of control following methamphetamine in Experiment 1 (A) or forced swim in Experiment 2 (B) (mean ± SEM). **Glucose levels were increased in MA-treated animals at 1 h following the first dose compared to SAL-treated animals (A). Glucose levels were decreased in forced swim animals at 7 h following the first swim compared to control animals (B). *p ≤ 0.05 *vs *control, ^†^p < 0.06 *vs *SAL.

For levels of glucose in blood, there was a Time main effect (p < 0.0001), however no main effect of Treatment was observed. There was also a nearly significant interaction of Treatment × Time, F (3, 76) = 2.63, p = 0.056. Analysis of the interaction revealed that MA-treated animals showed increased glucose levels 1 h after the first dose, but not thereafter (Fig. 3A).

#### Creatinine

**Figure 4:**
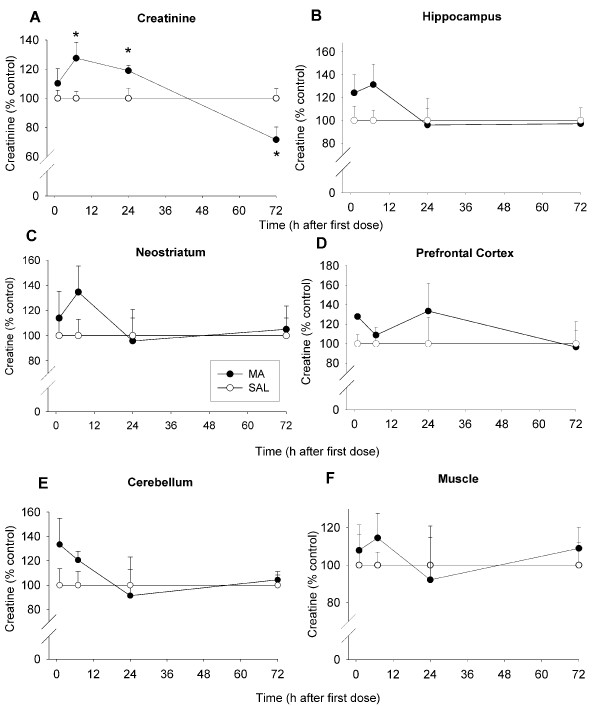
**Creatinine levels in plasma (A), creatine levels in brain (B-E), and muscle creatine (F) of animals in Experiment 1 (mean ± SEM expressed as a percent of control).** At 7 and 24 h, increases in creatinine were observed in MA-treated animals; at 72 h, a decrease in creatinine was observed in MA-treated animals (A). No differences in brain creatine in MA-treated animals compared to SAL-treated animals were observed in the hippocampus (B), neostriatum (C), prefrontal cortex (D), or cerebellum (E), at any of the time points examined. In addition, no differences were observed between MA-treated and SAL-treated animals in the levels of muscle creatine (F). * p ≤ 0.05 *vs *SAL.

There was a significant interaction of Treatment × Time on plasma creatinine, F(3, 77) = 5.81, p < 0.002, as well as the main effect of Time (p < 0.006). Creatinine was unchanged 1 h following the first dose of MA, however at 7 and 24 h, MA-treated animals showed increased creatinine compared to SAL-treated animals (Fig. 4A). At 72 h, MA-treated animals had decreased creatinine levels compared to SAL-treated animals (Fig. 4A).

#### Creatine

In addition to the plasma that was collected from groups of animals at 1, 7, 24, and 72 h after the first dose of MA, brain regions were also dissected from these animals and assessed for tissue creatine levels and monoamines. There were no significant effects of Treatment on creatine levels in any of the brain regions examined (hippocampus, neostriatum, prefrontal cortex, or cerebellum; Fig. 4B–E, respectively). In order to determine if creatine might have been altered in muscle at any of the time points, the gastrocnemius muscle was dissected and assessed for total creatine. Similar to brain, no effect of MA on creatine levels in muscle at any of the time points examined was found (Fig. 4F).

#### Monoamines

**Figure 5:**
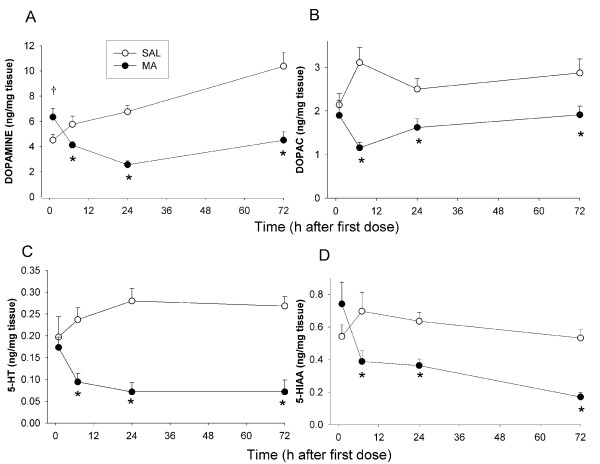
**Monoamine levels in the neostriatum in Experiment 1 (mean ± SEM). **MA-treated animals demonstrated decreases in DA (A), DOPAC (B), 5-HT (C), and 5-HIAA (D). Decreases were not observed 1 h after the first dose but became evident following the last dose of the MA treatment. *p ≤ 0.05 *vs *SAL, ^†^p < 0.07 *vs *SAL.

In the neostriatum, DA, DOPAC, 5-HT, and 5-HIAA were affected by Treatment, F(1, 68) = 29.51, 26.36, 44.90, and 12.68, respectively, p < 0.001, and this interacted with Time, Treatment × Time, for DA, DOPAC, 5-HT, 5-HIAA: F(3, 68) = 11.98, 2.84, 3.70, and 3.70, respectively, p < 0.05. Only DA and 5-HIAA showed a Time main effect (p < 0.01). Analysis of the interaction revealed that MA-treated animals showed decreased DA, DOPAC, 5-HT, and 5-HIAA at 7, 24, and 72 h after the first dose, but not at 1 h (Fig. 5A–D). For DA, levels at 1 h had a tendency to be higher in MA-treated animals compared to SAL-treated (Fig. 5A).

**Figure 6:**
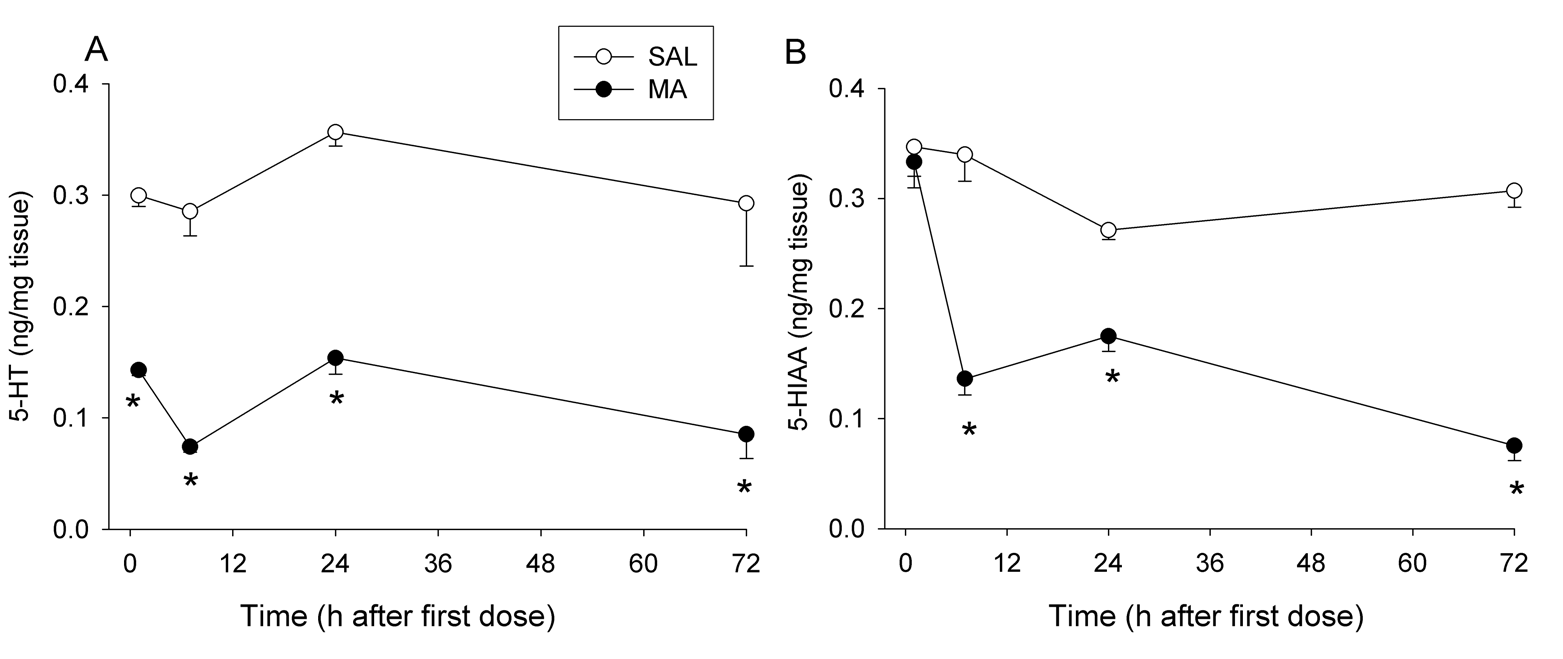
**Hippocampal monoamine levels in Experiment 1 (mean ± SEM).** MA-treated animals showed decreased 5-HT at 1, 7, 24, and 72 h (A) and decreased 5-HIAA at 7, 24, and 72 h (B). *p ≤ 0.05 *vs *SAL.

In the hippocampus, there were significant effects of Treatment, F(1, 68) = 155.67, p < 0.0001, and Time (p < 0.001) for 5-HT, but no interaction. MA-treated animals showed decreased 5-HT levels compared to SAL-treated animals at all time points (Fig. 6A). For 5-HIAA, there were significant effects of Treatment, F(1, 68) = 121.04, p < 0.0001, and Time (p < 0.0001). There was also an interaction of Treatment × Time for 5-HIAA, F(3, 68) = 14.94, p < 0.0001. MA-treated animals demonstrated decreased 5-HIAA except at 1 h after the first dose (Fig. 6B).

#### Glial Fibrillary Acidic Protein

**Figure 7:**
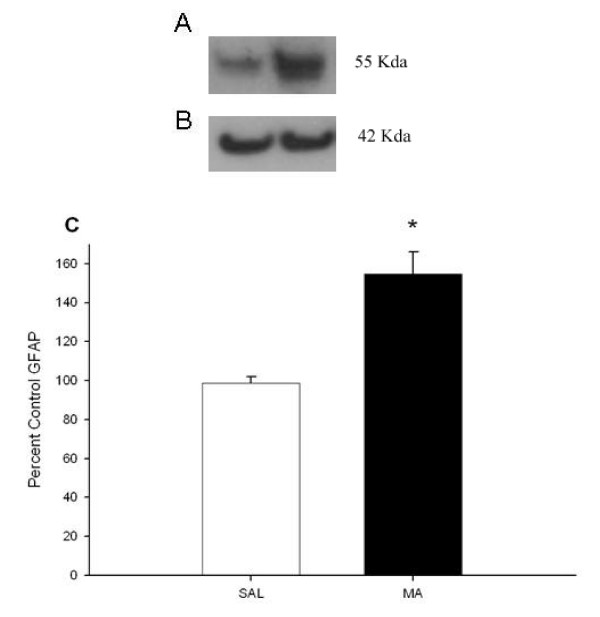
**GFAP levels 3 days following MA exposure by western blot analysis (mean ± SEM).** (A) GFAP was increased in MA-treated animals at 72 h following the first dose compared to SAL-treated animals (From Left to Right: SAL, MA). (B) No difference in actin levels was observed (From Left to Right: SAL, MA). (C) GFAP/actin shown as a percent of control (SAL, n = 3, MA, n = 4). Open bar = SAL, black bar = MA. *p < 0.05 *vs *SAL.

GFAP was measured in the neostriatum at 72 h by Western blot (Fig. 7A) and expressed against actin levels (Fig. 7B) to verify that the dosing regimen of MA used here produced neurotoxicity. MA-exposed animals showed significantly increased GFAP levels compared to SAL-treated animals, t(5) = -4.63, p < 0.01, which represented a 54% ± 12% increase in GFAP expression from MA treatment. Values are expressed as percent control (Fig. 7C).

#### Adrenal and Thymus weights

Since stress and/or high levels of circulating corticosterone can influence organ weights, we also collected the adrenals and thymuses to determine if changes in organ weights were occurring. The weights of the thymus and adrenal glands were analyzed for absolute tissue weight and as a percentage of body weight; for simplicity, only the percentage of body weight means are presented in Table 1. There were main effects of Treatment for the absolute weight and percentage of body weight of the thymus, F(1, 76) = 24.91 and 13.07, respectively, p < 0.001 (Table 1 for percent weight). There was also a main effect of Time (p < 0.0001), and an interaction of Treatment × Time, F(3,76) = 2.66, p = 0.05 for absolute values, but not for percentage of body weight. The effect of MA treatment was to reduce thymic weight. For the adrenal glands, a main effect of Treatment was observed for the absolute weight, F(1, 71) = 7.39, p < 0.01, but not for percentage of body weight. This effect was attributable to an overall decrease in adrenal weight in MA-treated animals compared to SAL-treated controls. There was also a main effect of Time for both absolute weight and percentage of body weight (p < 0.01), but no interaction of Treatment × Time (Table 1 for percentage of body weight).

**Table 1:** Adrenal and thymus weights of animals receiving saline (SAL) or (+)-methamphetamine (MA) in Experiment 1 or weights from control animals or animals forced to swim (FS) in Experiment 2 (Mean ± SEM)

**Tissue**	**Treatment**	**1 h**	**7 h**	**24 h**	**72 h**
Adrenal Gland (% Body Weight)	SAL	0.186 ± 0.007	0.214 ± 0.007	0.194 ± 0.011	0.151 ± 0.007
	MA	0.153 ± 0.008	0.187 ± 0.016	0.196 ± 0.014	0.168 ± 0.007
	Control	0.130 ± 0.014	0.194 ± 0.015	0.182 ± 0.014	-
	FS	0.151 ± 0.006	0.182 ± 0.011	0.188 ± 0.016	-
Thymus (% Body Weight)	SAL	2.314 ± 0.084	2.263 ± 0.175	1.481 ± 0.108	1.361 ± 0.076
	MA*	1.829 ± 0.125	2.041 ± 0.202	1.348 ± 0.084	0.9441 ± 0.071
	Control	1.786 ± 0.212	2.174 ± 0.123	1.640 ± 0.100	-
	FS	1.609 ± 0.129	2.057 ± 0.159	1.87 ± 0.075	-

*p < 0.05 vs SAL

### Experiment-2

Forced swim, a potent stressor in rats, was employed to determine if similar neurochemical markers were changed after stress as was seen after MA treatment.

#### Temperatures and Body Weights

Body temperatures during FS could not be obtained because of the animals being placed in water. There were no differences in body temperatures between treatments prior to FS testing (data not shown). Likewise, no differences in body weight were observed prior to FS (data not shown).

#### Corticosterone

As with MA administration, plasma was collected at 1, 7, and 24 h after the first of four 30 min forced swim sessions that were initiated at 2 h intervals. Corticosterone, glucose, and creatinine were assessed in the plasma. Plasma corticosterone was significantly affected by FS, Treatment, F(1, 41) = 111.40, p < 0.0001, and time of collection, Time, p < 0.0001. An interaction of Treatment × Time, F(2, 41) = 49.40, p < 0.0001, was also observed. At 1, 7, and 24 h after the beginning of the first swim, corticosterone levels were increased in FS animals compared to control animals (Fig. 2B).

#### Glucose

There were significant effects of Treatment, F(1, 41) = 7.34, p < 0.01, Time, p < 0.01, and the interaction of Treatment × Time, F(2, 41) = 5.36, p < 0.01. FS animals had significantly decreased plasma glucose 7 h after MA treatment compared to control animals; no differences in glucose levels were observed at 1 or 24 h (Fig. 3B).

#### Creatinine

**Figure 8:**
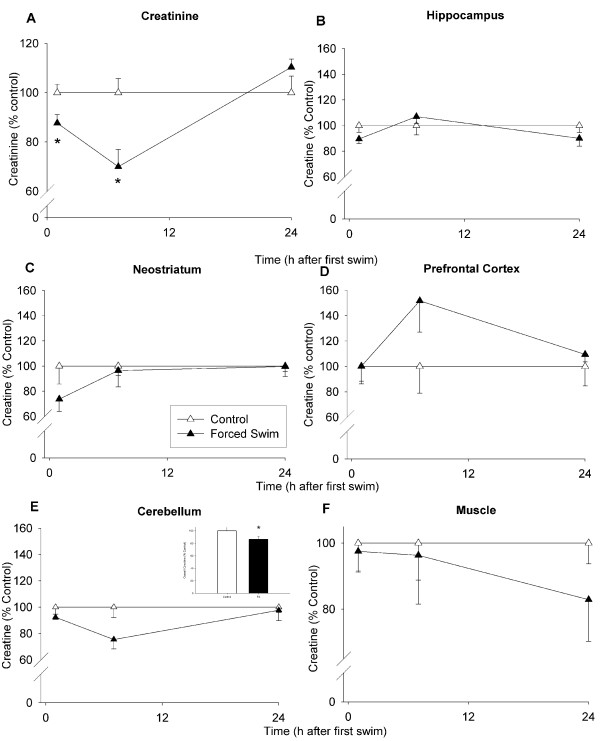
**Creatinine (A) levels in plasma, creatine levels in brain (B-E), muscle creatine (F) of animals in Experiment 2 (mean ± SEM expressed as a percent of control). **Forced swim animals demonstrated decreased creatinine levels compared to control animals at 1 and 7 h (A). No overall differences in creatine levels between forced swim and control animals were observed in hippocampus (B), neostriatum (C), or prefrontal cortex (D) In the cerebellum (E) there was a decrease in creatine (inset in E). No differences in muscle creatine levels were observed between forced swim and control animals (F). * p ≤ 0.05 *vs *control.

Levels of plasma creatinine were significantly altered by FS, Treatment, F(1, 41) = 7.65, p < 0.01, Time (p < 0.0001), and the interaction of Treatment × Time, F(2, 41) = 6.80, p < 0.01. FS animals showed decreased creatinine compared to control animals at 1 and 7 h after the first swim (Fig. 8A).

#### Creatine

As with animals that were treated with MA, the brains were concurrently removed after plasma collection and the hippocampus, neostriatum, prefrontal cortex, and cerebellum (Fig. 8B–8E, respectively) were dissected for examination of creatine and monoamines. For creatine, there was a Treatment main effect in the cerebellum, F(1, 25) = 5.15, p < 0.05, but no effect of Time, or the Treatment × Time interaction. FS animals showed decreased levels of creatine in the cerebellum compared to control animals (Fig. 8E inset). No differences were observed in the hippocampus, neostriatum, or prefrontal cortex (Fig. 8B–D, respectively). Similarly, for creatine levels in the gastrocnemius muscle, there were no significant effects of FS (Fig. 8F).

#### Monoamines

**Figure 9:**
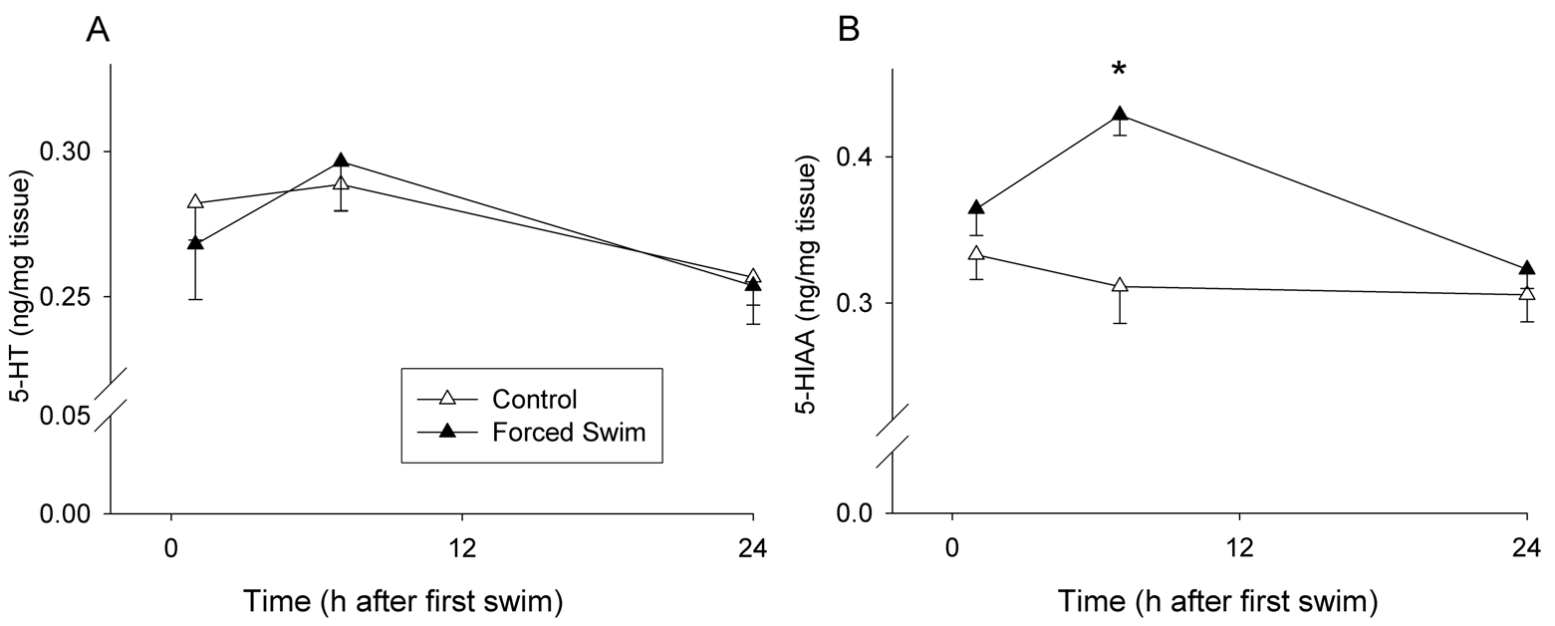
**Hippocampal monoamine levels in Experiment 2 (mean ± SEM).** No overall differences in 5-HT levels between forced swim and control animals were observed (A). Forced swim animals demonstrated increased 5-HIAA levels compared to control animals at 7 h (B). *p ≤ 0.05 *vs *control.

There was no Treatment effect of FS on DA, 5-HT, or their major metabolites in the neostriatum at 24 h (data not shown). Neostriatum for 1 and 7 h were unavailable because of a freezer malfunction. There was no effect of Treatment on 5-HT levels in the hippocampus (Fig. 9A), but there was a Time effect (p < 0.05). For 5-HIAA, there were significant effects of Treatment, F(1, 41) = 12.76, p < 0.001, Time (p < 0.05), and the interaction of Treatment × Time, F(1, 41) = 4.08, p < 0.05 (Control: 0.31 ± 0.03 ng/mg tissue and FS: 0.43 ± 0.01 ng/mg tissue). 5-HIAA was increased in FS animals compared to controls, although this was significant only at the 7 h time point (Fig. 9B).

#### Adrenal and Thymus Weights

There were no significant effects of FS on either absolute or percentage of body weight for adrenal or thymic weights at any of the time points examined (Table 1).

## Discussion

In the present experiments we compared the effects of MA and forced swim on corticosterone, energy homeostasis, and neurotransmitters to determine if the effects of MA were unique to MA or if the effects could be characterized as a general stress response. In this regard, both MA and forced swim induced release of corticosterone from the adrenal gland, however forced swim produced greater levels of corticosterone than MA. Interestingly, only MA produced reductions in the weights of the adrenal glands and thymuses, even though forced swim had the larger effect on corticosterone. Both MA and forced swim had effects on glucose that were short-lived and time- and treatment-dependent since increases were observed with MA while decreases were observed with forced swim. Conversely, for plasma creatinine, MA produced an increase during the first 24 h, whereas a reduction was observed during at least the first 7 h of forced swim. Forced swim induced only a transient increase in 5-HIAA in the hippocampus, however MA produced pronounced changes in monoamines in the neostriatum and hippocampus. It appears that the hippocampus may be more sensitive to the effects of MA, since 5-HT in this region was already showing a reduction in levels 1 h after a single dose. Although creatine levels appeared slightly affected by MA, only forced swim caused a significant change in creatine and only in the cerebellum. Taken together, these data demonstrate that MA has a unique profile of effects on hormone release, glucose homeostasis, creatinine, and monoamines that cannot be explained by a general stress response.

The hypothalamic-pituitary-adrenal (HPA) axis is a homeostatic system activated by both psychological and physiological stressors that induces a cascade of neurotransmitter and hormone release [18]. Not only is the HPA axis activated during stress, it also participates in regulation of homeostatic processes such as digestion, immune responses, and energy utilization [18]. HPA axis activation ultimately leads to the release of glucocorticoids, namely corticosterone in the rat, which induces immediate responses such as increased availability of glucose (via gluconeogenesis) and inhibition of immune, reproductive, and digestive processes; all with the purpose of facilitating the coping, adaptation, and recovery from the stressor, although prolonged exposure can be detrimental [18-21]. In this study, we found similarities between MA treatment and forced swim with regard to corticosterone such that each treatment caused prolonged activation of the adrenal gland. These data suggest that other hormones of the HPA axis such as ACTH or corticotrophin-releasing factor should be examined in future studies to determine if the long-term increases in corticosterone are a direct effect of MA on the adrenal or continued activation of the HPA axis. Considering the half-life of MA in rats is approximately 1 h [22], the 72 h time point exceeds the half-life by > 60 times, indicating that no detectable MA would be present and suggesting that the HPA axis is likely altered. These long-lasting alterations to the adrenal glands, thymus, and corticosterone may indicate a cumulative negative effect of MA or forced swim on the regulation of many of stress-related components and their receptive tissues ("allostatic load") [23]. In addition, the alterations in corticosterone may represent a shift in baseline levels that alters regulatory mechanisms and the ability to maintain homeostasis during additional stressors or change ("allostasis") [23]. Alternatively the prolonged corticosterone increase after the treatment period may be the result of an altered circadian response, and previous studies have demonstrated that MA treatment can act as a zeitgeber and alter circadian rhythms [24]. However, it seems unlikely that changes in circadian patterns alone could explain the long lasting elevation in corticosterone and this does not explain the increased levels observed with forced swim. Investigation of a circadian effect was outside of the scope of the present study. Finally, since stress activates and modulates many other physiological pathways and organs, such as the thyroid, and these may also produce changes in learning and memory, a profile of these hormones could provide useful information. Likewise, the forced swim paradigm used here might profitably be examined for its impact on learning and memory.

While both MA and FS produced large and protracted increases in corticosterone, other measures were different between the two experimental conditions. For example, MA treatment resulted in an immediate increase (1 h) in plasma glucose, while FS treatment caused opposite effects, a decrease at 7 h. Others have also shown that MA treatment increases glucose levels in fetal sheep up to 120 min after maternal administration [25]. The decrease in glucose in FS animals was most likely due to the utilization of available glucose during swimming, as levels returned to baseline by 24 h. After MA treatment, creatinine levels in plasma were increased at 7 and 24 h, but decreased at 72 h whereas after FS treatment they were decreased at 1 and 7 h and returned to control levels by 24 h. The degradation of creatine to creatinine is a concentration-dependent, nonenzymatic process and plasma creatinine levels are often used as markers for kidney function [16]. In this study, possibilities for increased creatinine after MA administration would most likely be secondary to rhabdomyolysis or renal dysfunction as has previously been observed in human MA users [5]; however, renal function was not evaluated in this study. While certain effects observed in humans following MA were observed in the present model, others, such as altered creatine in the brain were not.

Creatine levels were not altered in MA-treated animals, but FS-treated animals demonstrated an overall decrease in creatine in the cerebellum, further demonstrating that the effects of MA are not related to a general stress response. The cerebellum is highly involved with motor output and increased activity of this brain region during FS may have resulted in the decreased creatine. While we chose a stressor where muscle activity was present, FS is likely to have exaggerated the motoric burden of the animals. It should be noted that altered brain creatine levels in humans have been shown after long-term use and abstinence from MA, whereas this study examined the acute effects of MA; human studies on acute effects are unknown. The present experiment did not evaluate the levels of creatine in relation to other compounds such as phosphocreatine or N-acetyl acetate that are obtained during spectroscopy and therefore we cannot rule out that changes in the ratio of these compounds might have occurred. Nonetheless, others have shown that phosphocreatine in the brain is unaltered following various doses of (+)-amphetamine or (-)-MA [26-28].

MA treatment resulted in the expected astrogliosis as shown by increased GFAP expression 72 h after treatment in the neostriatum and decreased monoamines in the neostriatum and hippocampus. Only a transient increase in 5-HIAA was observed following forced swim. This pattern is consistent with the well-described neurotoxic profile of MA treatment [12,29-31]. Decreases in monoamines were observed starting at 7 h after the first dose of MA and remained decreased up to72 h. Most studies have examined the long-term effects of MA on monoamine levels (3 days to months after treatment), although striatal DA has previously been shown to be depleted as early as 36 h after the first dose of repeated MA treatment and tyrosine hydroxylase (TH) activity decreased as early as 8 h after one 15 mg/kg dose of MA [32]. The present data are the first to show reduced DA as early as 7 h after the first dose. Tryptophan hydroxylase (TPH) activity in the striatum has been demonstrated to be decreased as early as 3 h after a single 10 mg/kg dose of MA [33] and therefore, the reduction in TPH prior to decreased 5-HT is consistent. Hippocampal 5-HT and 5-HIAA have also been shown to be decreased as early as 36 h after the first dose of a repeated MA treatment [34].

## Conclusion

These data provide verification that the treatment regimen for MA used here was effective at inducing multiple indices of MA-induced neurotoxicity. Unlike human abusers, creatine was unaltered in rats, although this may be related to duration of use, since treatment was limited here to a single day. Since humans show reductions in monoamines after long-term use, an increase in creatinine [5] and cortisol levels [4] during MA intoxication, the dosing model used here appears to mimic certain effects observed following human use and abuse. However, the lack of effect on creatine suggests that longer exposure periods in rats may be required to fully demonstrate the effects observed in humans. A longer dosing period would likely have effects on the behavioral consequences observed in rats as well and should be the subject of future investigations. Forced swim caused similar or even higher increases in corticosterone than MA, yet did not induce other changes similar to MA, indicating that the effects of the drug are not attributable to its stress-like effects. Overall, the data suggest that MA induces a unique pattern of effects that cannot be explained by a general stress phenomenon. The present data provide a more complete picture of the short-term effects of MA and the similarities and dissimilarities to multiple forced swims.

## Methods

### Animals and conditions

Male Sprague-Dawley rats (250–275 g; Charles River Laboratories, Raleigh, NC) were allowed to acclimate to housing conditions (19 ± 1°C, 50 ± 10% humidity, and 14 h light: 10 h dark cycle with lights on at 600 h) for 2 days prior to either MA or forced swim treatment. Rats were first housed in the colony room in pairs in cages measuring 46 × 24 × 20 cm, but separated on the day of testing to 28 × 16 × 12 cm polycarbonate cages in a different room maintained at an ambient temperature of 24°C during drug or forced swim administration. Separation of animals was done to ensure a consistent environment rather than have the potential for aggression when MA was administered, especially since this would influence any stress-related parameters. Food and water were provided ad libitum except during treatment. All procedures were conducted in accordance with the National Institutes of Health *Guidelines for the Care and Use of Laboratory Animals*, and were approved by the Institutional Animal Care and Use Committee of Cincinnati Children's Research Foundation. The vivarium was accredited by the Association for the Assessment and Accreditation of Laboratory Animal Care (AAALAC).

### Experiment-1

(+)-Methamphetamine-HCl (expressed as the freebase, from the National Institute on Drug Abuse and greater than 95% pure) or isotonic saline (SAL) was injected subcutaneously to animals in 4 doses with a 120 min inter-dose interval beginning at 900 h. Each dose consisted of 10 mg/kg in a volume of 3 ml/kg for a total daily dose of 40 mg/kg over the 6 h period. Each animal was weighed prior to the first injection. For Experiment-1, the treatment groups were MA and SAL and were run in two separate cohorts. Four post-treatment time points were assessed: 1, 7, 24, and 72 h after the first injection (n = 8, 8, 17, and 10, respectively). The 72 h time point was included to demonstrate neurotoxicity with this dosing regimen, although it was the early time points that were of interest. Injections of both MA and SAL were delivered in the dorsum and injection sites were varied to prevent skin irritation.

### Experiment-2

Forced swim has previously been demonstrated to increase corticosterone levels more than other stressors [17]. Forced swim for 30 min was performed by placing each rat in a 15 cm (diameter) by 46 cm tall PVC cylinder filled with 35 cm of water (22 ± 1°C) either once or four times; as with MA treatment. FS was administered beginning at 900 h for the single stress group and beginning at 900 h × 4 at 2 h intervals for the multiple FS group. Animals were weighed prior to the beginning of the FS regimen. Three time points were examined: 1, 7, and 24 h after the beginning of the first FS. Group sizes were FS = 8, control = 8 for each of the 3 time points, or 48 animals total. Between FS trials, all animals were maintained in 28 × 16 × 12 cm polycarbonate cages in a separate room from the colony, exactly as in Experiment-1.

### Body Temperature

On the day of arrival, animals were lightly anesthetized with isofluorane and injected with implantable temperature transponders (IPTT-300: Bio Medic Data Systems, Seaford, DE). The subcutaneous probes were used to alleviate the hyperthermia and stress of rectal temperature measurements during dosing [35] and the physical manipulation of the animal that occurs with such recordings. Previous data have demonstrated that handling and rectal temperature measurements increase corticosterone and body temperature [36].

During the period of MA or FS treatment, body temperatures were monitored every 30 min beginning with the first treatment and for the next 8 h. In order to prevent excessive hyperthermia during MA treatment, a cooling protocol was followed. If an animal's temperature reached 40°C it was placed in a shallow bath of room temperature water and its temperature was then monitored every 5–10 min. Once an animal's temperature fell below 40°C it was removed from the water and returned to its holding cage. After 8 h (2 h after the last dose), animals were returned to the colony room. A comparison of cooled animals versus non-cooled animals demonstrated that cooling did not significantly attenuate the depletions in monoamines.

### Body weight

Animals were weighed on the day of drug or FS administration prior to the first treatment. For experiment-1, animals were also weighed 24, 48, and 64 h after MA dosing.

### Tissue collection

At the designated time points, animals were transported individually to an adjacent suite (< 30 s after removal from cage), decapitated [37], and blood collected in polyethylene tubes (12 × 75 mm) containing 2% EDTA (0.05 ml/tube). The brain was rapidly removed, placed over ice, and the neostriatum and hippocampus were dissected as described previously [38] as well as the prefrontal cortex and cerebellum. All tissues were frozen on dry ice and stored at -80°C until assayed.

The adrenal glands and thymus from each animal were removed, freed of fatty tissue, and weighed. The data for tissue were expressed as absolute tissue weight or as a percentage of body weight. For experiments-1 and 2, initial body weights for percentage of body weight analysis were utilized except for experiment-1 the 64 h body weight was used for the 72 h time point. These tissues were collected since they are known to be affected by hormones of the HPA axis, particularly during increased activation of this pathway [39-42].

### Corticosterone, creatinine and glucose determinations

Blood was collected in ice-chilled polypropylene tubes containing EDTA, centrifuged at 4°C for 15 min, plasma aliquoted, and stored at -80°C until assayed for corticosterone and creatinine (Crn). For the assessment of corticosterone, plasma was diluted 5:1 in assay buffer and assayed in duplicate using a commercially available EIA kit for corticosterone (IDS, Fountain Hills, AZ). Glucose in whole blood was determined using a commercially available glucometer (Precision Xtra, Abbott Laboratories, Bedford, MA).

For creatinine, 300 μl of methanol were added to 100 μl of plasma in microcentrifuge tubes, vortex-mixed for 1 min, and centrifuged at 9,400 × g for 10 min. The supernatant was transferred to an autosampler vial, and 50 μl was automatically injected into a high pressure liquid chromatography (HPLC) system. A Rainin Microsorb-MV reversed-phase column (4.6 mm × 15 cm, 5-μm bead size) was used. The mobile phase consisted of potassium phosphate monobasic (40 mmol/l), sodium dodecyl sulfate (10 mmol/l), 190 ml methanol, and 180 ml acetonitrile (adjusted to a pH of 3 with phosphoric acid). The flow rate was 0.9 ml/min and the retention time for creatinine and cimetidine (internal standard) were 6.9 min and 11.4 min, respectively. The absorbance detector was set at 236 nm. The analysis was run at 30°C.

### Creatine assessment

Free creatine was measured in the gastrocnemius muscle and in several brain regions including: cerebellum, neostriatum, prefrontal cortex, and hippocampus. Gastrocnemius muscle was chosen because MA-treated animals display hyperactivity and FS induces vigorous paddling, both behaviors require the use of the gastrocnemius muscle. Free creatine was measured using a fluorimetric technique described previously [43] with modification. Briefly, this method combines ninhydrin, at alkaline pH, with guanidine, and monosubstituted guanidines to form a fluorescent product that is specific for creatine. The assay has an effective range from 1.0 × 10^-7 ^M to 2.5 × 10^-5 ^M. The creatine standard was made from reagent grade creatine (MP Biomedicals, Inc.). Each sample was weighed and 200 μl of RIPA (50 mM Tris, 1% NP-40, 25% Na-deoxycholate, 150 mM NaCl, 1 mM EDTA, Na_3_VO_4_, 1 mM NaF along with 1 μg/ml of each: aprotinin, leupeptin, pepstatin) for each 100 mg of tissue was added prior to tissue homogenization. Homogenates were heated for 5 min at 103°C and then centrifuged at 32,091 × g (4°C) for 10 min. The supernatant was then collected.

Creatine determination involved adding 120 μl of the supernatant with 240 μl of Ba(OH)_2 _and 240 μl of ZnSO_4_. After the formation of a precipitate the samples were centrifuged at 32,091 × g (4°C) for 3 min. A total of 150 μl of the supernatant was placed in a 96-well, black-bottomed plate (ISC Bioexpress) and each sample was run in triplicate. Ninhydrin (75 μl) was added to each well while keeping the plate protected from external light during and after the addition of 75 μl of KOH. Eight min following the addition of the KOH, the plate was read using a fluorimeter (Spectramax M2; Molecular Devices) at an absorbance of 410 nm excitation and 525 nm emission. Flourimetric readings were recorded and creatine concentrations were calculated from standard curves generated from each plate and expressed as μmol/mg tissue.

### Monoamine assessment

Brain tissue concentrations of dopamine (DA), 3,4-dihydroxyphenylacetic acid (DOPAC), serotonin (5-HT), and 5-hydroxyindolacetic acid (5-HIAA) in the neostriatum and 5-HT and 5-HIAA in the hippocampus were quantified using HPLC with electrochemical detection as described previously [44]. Tissues were homogenized in 50 volumes of 0.2 M perchloric acid and centrifuged for 6 min at 10,000 × g. Aliquots of 20 μl were injected onto a C18-column (MD-150, 3 × 150 mm; ESA, Chelmsford, MA) connected to either a LC-4B amperometric detector (Bioanalytical Systems, West Lafayette, IN) or a Coulochem (25A, Chemsford, MA) detector and an integrator recorded the peak heights that followed each injection. The potential for the LC-4B was 0.6 V and the potentials of E1 and E2 on the analytical cell (model 5014B) of the Coulochem were -150 and -160 mV, respectively and Ag/AgCl reference electrodes were used. The mobile phase consisted of 35 mM citric acid, 54 mM sodium acetate, 50 mg/l of disodium ethylenedeamine tetraacetate, 70 mg/l of octanesulfonic acid sodium salt, 6% (v/v) methanol, 6% (v/v) acetonitrile, pH 4.0, and pumped at a flow rate of 0.4 ml/min. Quantities of the analytes were calculated on the basis of known standards. Retention times for DOPAC, DA, 5-HIAA, and 5-HT were approximately 3.0, 5.0, 7.0, and 20.0 min, respectively.

### GFAP Analysis

Neostriata from Experiment-1 (72 h) were homogenized in cold RIPA buffer. Protein concentrations were determined using Pierce BCA Protein Assay Reagent Kit (Rockford, IL) according to the manufacturer's specifications and homogenates were diluted to a concentration of 1 μg protein/ml with PBS. Samples (10 μl) were diluted 1:1 with 2× loading buffer, boiled for 5 min and loaded on 8–16% polyacrylamide gels (ISC BioExpress, Kaysville, UT) with control and experimental animals equally distributed on each gel. Proteins separated by electrophoresis were transferred in a buffer of 25 mM bicine, 20 mM Tris, and 10% MeOH at 40 V for 1.5 h to Immobilon-PVDF membranes (Millipore, Bedford, MA). Membranes were blocked by incubating at 4°C with 3% BSA in PBS with 0.1% Tween-20 (PBST) overnight to prevent nonspecific binding of antibodies. Membranes were then incubated for 1 h with a 1:500 dilution of GFAP (Fitzgerald, Concord, MA) or actin (Chemicon International, Temecula, CA) antibody. Following washing (3 × 5 min with PBST), membranes were incubated for 30 min at room temperature with 1:5000 dilution of goat anti-mouse conjugated to alkaline phosphatase for both GFAP and actin. Membranes were washed (3 × 5 min with PBS), followed by incubation with CDP Star chemiluminescent (KPL, Gaithersburg, MD) substrate for 5 min. Membranes were exposed to film until adequate signal development was achieved; films were then scanned and protein bands were quantified using ImageJ software (NIH, Bethesda, MD). The density of the GFAP or actin band of the treated samples was divided by the density of the control sample in each gel and GFAP values were divided by actin values to control for protein concentration for each sample.

### Data Analysis

Data were analyzed using factorial analysis of variance (ANOVA), general linear model (GLM; SAS Institute, Cary, NC) or t-tests (GFAP only). Treatment (MA or SAL, Exp-1; FS or Naïve, Exp-2) and Time (1, 7, 24, or 72 h) were between-subject factors. Significance was set at a level of *p *≤ 0.05. Data are presented as group means ± SEM.

## Abbreviations

5-HIAA: 5-hydroxyindolacetic acid; 5-HT:  5-hydroxytryptamine; ACTH: adrenocorticotrophin hormone; ANOVA: analysis of variance; ATP: adenosine triphosphate; DA: dopamine; DOPAC:  3,4-dihydroxyphenylacetic acid, FS: forced swim; GFAP: glial fibrillary acidic protein; HPA: hypothalamic-pituitary-adrenal; HPLC: high pressure liquid chromatography; MA: methamphetamine; SAL: saline.

## Competing interests

The authors declare that they have no competing interests.

## Authors' contributions

NRH was involved in all aspects of the study from conception to manuscript preparation. TLS and MRS participated in data acquisition including but not limited to brain dissections and western blot analysis. PHT, JPL, and GAG participated in HPLC collection and interpretation of neurochemical data. CVV and MTW conceived and designed the study as well as analyzed and interpreted the results, and revised the manuscript. All authors approved the final manuscript.
